# Is the effect of menu energy labelling on consumer behaviour equitable? A pooled analysis of twelve randomized control experiments

**DOI:** 10.1016/j.appet.2023.106451

**Published:** 2023-01-05

**Authors:** Eric Robinson, Emma Boyland, Paul Christiansen, Ann F. Haynos, Andrew Jones, Una Masic, Deirdre Robertson, Katy Tapper, Lucile Marty

**Affiliations:** a Department of Psychology, University of Liverpool, Liverpool, UK; b Department of Psychiatry and Behavioral Sciences, University of Minnesota, Minneapolis, MN, USA; c Gender Identity Development Service, Tavistock and Portman NHS Trust, London, UK; d Behavioural Research Unit, Economic and Social Research Institute, Ireland & School of Psychology, Trinity College Dublin, Dublin, Ireland; e Department of Psychology, City, University of London, UK; f Centre des Sciences Du Goût et de l’Alimentation, CNRS, INRAE, Institut Agro, Université Bourgogne Franche-Comté, F-21000, Dijon, France

**Keywords:** Energy labeling, Calorie labels, Individual differences, Obesity policy

## Abstract

Menu energy labelling has been implemented as a public health policy to promote healthier dietary choices and reduce obesity. However, it is unclear whether the influence energy labelling has on consumer behaviour differs based on individuals’ demographics or characteristics and may therefore produce inequalities in diet. Data were analysed from 12 randomized control trials (N = 8508) evaluating the effect of food and drink energy labelling (vs. labelling absent) on total energy content of food and drink selections (predominantly hypothetical) in European and US adults. Analyses examined the moderating effects of participant age, sex, ethnicity/race, education, household income, body mass index, dieting status, food choice motives and current hunger on total energy content of selections.

Energy labelling was associated with a small reduction (f^2^ = 0.004, −50 kcal, p *<* 0.001) in total energy selected compared to the absence of energy labelling. Participants who were female, younger, white, university educated, of a higher income status, dieting, motivated by health and weight control when making food choices, and less hungry, tended to select menu items of lower energy content. However, there was no evidence that the effect of energy labelling on the amount of energy selected was moderated by any of the participants’ demographics or characteristics. Energy labelling was associated with a small reduction in energy content of food selections and this effect was similar across a range of participants’ demographics and characteristics. These preliminary findings suggest that energy labelling policies may not widen existing inequalities in diet.

## 1. Introduction

The development and rapid growth of the obesity ‘crisis’ has coincided with a societal shift towards consuming more energy dense foods and drinks that are prepared, sold and consumed outside of the home (Popkin et al., 2012). The nutritional quality of food and drink sold in the out of home food (OOHF) sector is often lower in nutritional quality and higher in energy (Roberts et al., 2018; Robinson et al., 2018), compared to food prepared in the home. To address this, a number of countries, including the US, England and parts of both Australia and Canada, have introduced policies that require large OOHF sector chains to label menus with product energy (kcal) content (Cleveland et al., 2018; Robinson et al., 2021a).

Recent reviews of research examining the impact of menu energy labelling on consumer behaviour have concluded that energy labelling has either a small or no overall influence on the total amount of energy customers order and consume in the OOHF sector (Bleich et al., 2017; Crockett et al., 2018). In line with this interpretation, some studies have found a modest reduction in energy selected attributable to energy labelling, whilst other studies have found no effect of labelling (Liu et al., 2012; Marty et al., 2021a). One potential explanation for these heterogenous findings is that the influence energy labelling has on consumer behaviour differs based on population characteristics. Yet, systematic reviews have concluded that there is an absence of evidence on whether any impact energy labelling has on consumer behaviour is moderated by demographics or characteristics such as sex, age or Body Mass Index (BMI) (Bleich et al., 2017; Crockett et al., 2018). It will be particularly important to understand whether energy labelling policies benefit those who are in most need of intervention.

A relatively small number of individual studies have explored demographic patterning of self-reported use of energy labelling in the OOHF sector using observational study designs. For example, Chen et al. examined the proportion of diners at restaurants in the US reporting using energy labelling and found that women and higher income groups were more likely to report using labelling when making food choices (Chen et al., 2015). Conversely, Pulos and Leng adopted a similar approach and found no evidence that women were more likely to report using energy labelling, but did find some evidence that younger adults were more likely to report using energy labelling than older adults (Pulos & Leng, 2010). Although these findings are suggestive that the influence energy labelling may be moderated by individuals’ characteristics, a significant limitation of these studies is that self-reported energy labelling use is likely to be prone to bias (Herbert et al., 1995) (e.g. over-reporting of usage in some demographic groups). Furthermore, studies have been observational and this does not permit causal inference on the influence of energy labelling (e.g., whether the effect of energy labelling on energy purchased is larger among females than males). A number of studies have adopted randomized controlled trial (RCT) designs to experimentally examine the causal impact of energy labelling (vs. absence of energy labelling) on food choice (Robinson et al., 2021a). However, few of these studies have examined whether the effect of energy labelling on consumer behaviour is moderated by participant demographics or characteristics. Furthermore, when moderation by participant characteristics has been explored only a limited number have been examined (Roberto et al., 2010).

Given that energy labelling policies in the OOHF sector are now considered a viable policy option to address obesity (Robinson et al., 2021a), it will be important to understand whether the impact of this strategy differs based on participants’ demographics or characteristics. In the present research, we compiled data from 12 RCTs that used similar methods to examine the effect of energy labelling on consumer behaviour and measured a range of participant level characteristics. Our aim was to explore whether participant level characteristics moderate the effect of energy labelling on consumer behaviour.

## 2. Methods

### 2.1. Overview of studies

In the present research, we combined data from 12 between-subject (parallel arms) RCTs in which participants made non-alcoholic food and/or drink choices on menus after being randomly allocated to presence vs. absence of energy labelling information (Haynos & Roberto, 2017; Marty et al., 2020, 2021a; Masic et al., 2017; Robertson & Lunn, 2020; Tapper et al., 2022; VanEpps et al., 2021). Studies were conducted in Europe (n = 6) and North America (n = 6). The majority of studies sampled from the general population (n = 8) and a minority recruited predominantly from university campuses (n = 4). The majority of studies were conducted online (n = 9, as opposed to in laboratory conditions, n = 3) and n = 11 studies involved participants making simulated dietary choices (i.e. participants were asked to make hypothetical food/drink selections), whereas a single study also involved participants consuming their chosen meal (Robertson & Lunn, 2020). Participants were typically shown menus with food and/or drink items and asked to select which item(s) they would choose, allowing for total energy content of all food/drink chosen to be examined. In most studies participants were required to select a meal, although in two studies participants were asked to select a drink (and could also select accompanying food items, if desired) (Tapper et al., 2022) and in one study participants were asked to choose between pairs of food items (Masic et al., 2017). Studies are described in details in Table 1. We included all published studies on menu energy labelling conducted by members of the research team which used an experimental design and measured a minimum of one participant characteristic.

### 2.2. Energy labelling

Participants made food/drink choices in the presence vs. absence of energy labelling and, in all studies, energy content of food/drink options was presented in kcal next to each food/drink option. In some studies participants made food/drink choices in the presence of more than one type of energy labelling condition (e.g. kcal labelling only, kcal labelling plus physical activity equivalence information). To ensure consistency across studies, we only included the energy labelling condition from each study that most closely resembled current US and England usage of energy labelling in the OOHF sector (e.g. energy information equal in size to price presented to the right of menu item and/or next to item price, daily energy requirements for adults also present).

### 2.3. Participant characteristics

Participants self-reported demographic and personal characteristics. To ensure sufficient analytic sample sizes, we only included participant characteristics that were measured in at least three studies and we defined a participant characteristic as any measurement of interindividual difference that relates to demographic (e.g. age), trait (e.g. being the type of person that is motivated by health when making food choices) or state (e.g. current hunger) characteristics. Participant sex and age were available in all studies. BMI was available in 10 studies (8 studies used participant reported weight and height, 2 studies used researcher measurement). Highest education level achieved (6 studies), household income (6 studies) and ethnicity/race (5 studies) were also commonly available. Of the 12 studies, 9 measured whether participants were currently dieting vs. not. Six of the 12 studies included self-reported measures on the extent to which day-to-day food choices were motivated by health and weight control. Finally, 5 studies measured participant hunger levels during the study (e.g. to control for hunger or examine whether energy labelling was more/less influential when very hungry).

### 2.4. Data treatment

For analytic purposes, sex was categorised as male vs. female (due to there being too few participants selecting other sex-based response options across studies). Age (18–25, 26–34, 35–50, >50 years) and BMI (<18.5, 18.5–24.9, 25–29.9, >30 kg/m^2^) were treated as categorical variables. We excluded participants with implausible weight (<30 kg or >250 kg) and height (<145 cm or >3 m) values, or likely implausible BMI (<12.5, >70) values (Robinson et al., 2021b). Consistent with the included studies, highest education level qualification was characterised as university level vs. less than university level. Household income was available for 2 UK studies and 4 US studies. To account for country-level differences, household income was examined by quintile (based on quintiles of the UK and US studies data separately). For income data, if a participant reported a household income that appeared extreme and therefore likely implausible (i.e. > 10 times the UK median equivalised income [>£300,000] or US median [>$650,000] their data were treated as missing for analyses involving income. Consistent with how ethnicity/race was reported in studies, and due to limited sample size for individual ethnic minority groups, ethnicity/race was treated as white vs. non-white ethnic minorities. Current dieting status was categorical (currently dieting vs. not, as measured in included studies). The extent to which participants reported being motivated by health when making day-to-day food choice was measured on a 1–7 scale, as was motivation based on weight control, adopting the widely used Food Choice Questionnaire (Steptoe et al., 1995). As in previous research (Robinson et al., 2017), hunger was measured on a 0–100 scale visual analogue scale. Food choice motives and hunger were therefore treated as continuous variables for analytic purposes. We planned to only use data from the primary analyses of the original published studies (e.g. if a study excluded participants from analyses based on having identified the aims of the study, then we also excluded these participants).

#### 2.4.1. Analyses

##### 2.4.1.1. Main analyses.

See https://osf.io/28cxt/for the pre-registered analysis protocol in full. The dependent variable in all analyses was total energy selected in kcal (Z-scored on a study-by-study basis to standardise the values across studies). Linear mixed effects models (with a random effect of study)^1^ were used and the primary model included independent variables for which data was available from all studies (labelling condition (present vs. absent), sex and age entered in a first model step). In the second step of the model, interaction terms between participant characteristics (sex, age) and labelling condition were entered. We also repeated the above primary analysis with unstandardized total energy selected as the dependent variable to confirm results were consistent when combining raw data from studies. To maximise sample size, we then ran separate models for all other participant characteristics variables by adding them to the primary model (additional models). We examined whether their inclusion (main effect) and interaction effect with labelling condition at the second step of the primary model increased variance explained. Because the health and weight control food motivation measures came from the same 6 studies, they were entered into the same model.

##### 2.4.1.2. Planned sensitivity analyses.

We repeated the main analyses with age and BMI treated as continuous variables. We also repeated the main analyses when excluding Masic et al. (Masic et al., 2017) as in this study participants chose from pairs of food options from a menu, as opposed to ordering from a menu.

##### 2.4.1.3. Unplanned sensitivity analyses.

One study in which sex was measured and one study in which education level was measured recruited females only and participants with a university degree only, respectively. We therefore examined if results relating to sex and education level remained the same with these studies excluded.

##### 2.4.1.4. Statistical power and sample size.

Due to the large number of comparisons and models being conducted (interactions between 10 predictor variables and labelling condition, 0.05/10), alpha was set at p < 0.005 across all analyses. Across models, to detect statistically small increases in variance explained (f^2^ = 0.02, 80% power, p < 0.005) we estimated that a minimum N = 944 was required, indicating that we were well powered in all models conducted. See online supplementary materials for detailed power calculation information.

## 3. Results

### 3.1. Sample characteristics

Across the 12 studies, the final sample included a total of 8508 participants. Table 2 presents sample characteristics in full. The mean age was 36 years (SD = 14.8). The sample consisted of more females (58%) than males, more highly educated participants (62% with university degree or higher) and was predominantly white in ethnicity/race (81%). Mean BMI was 26.6 kg/m^2^ (SD = 6.5) and 29% of samples were in the normal (18.5–24.9) BMI range. The majority of participants were not currently dieting (75%).

### 3.2. Moderation of effects of energy labelling on total energy selected by age and sex

In the primary model testing the effect of labelling, sex and age on total energy selected (Table 3, Primary model – step 1) parameter estimates indicated that lower amounts of energy were selected in labelling condition (−0.13, SE = 0.02, 99.5%IC [−0.19; −0.07]), by female participants (−0.24, SE = 0.02, 99.5%IC [−0.30; −0.18]) and by older participants (35–50 vs. 18–25: −0.13, SE = 0.03, 99.5%IC [−0.21; −0.04] and >50 vs. 18–25: −0.42, SE = 0.03, 99.5%IC [−0.49; −0.31]). However, interactions between labelling condition and sex or age were not significant (Table 3, Primary model – step 2). This pattern of results was confirmed in a sensitivity model when age was treated as continuous variable (Table 3, Sensitivity model 1), in a sensitivity model excluding participants from Masic et al. (Table 3, Sensitivity model 2) and in a sensitivity model excluding Haynos et al. where only female participants were included (Table 3, Sensitivity model 3). Although in the primary model the effect of energy labelling condition was statistically significant, it was very small in magnitude (f^2^ = 0.004, equivalent of R^2^ = 0.004 or 0.4% of variance explained) when compared to effect size guidelines for Cohens f^2^ (f^2^ small = 0.02, medium = 0.15, large = 0.35) (Selya et al., 2012). In the model on unstandardized (raw) total kcal selected, lower amounts of energy were selected in the labelling condition (−50.3 kcal, SE = 11.4, 99.5%IC [−82.4; −18.3]) (Table 3, Primary model raw). Consistent with the main analyses, interactions between labelling condition and sex or age were non-significant. See online supplementary materials for individual study data (energy selected) by condition.

### 3.3. Moderation by other participant characteristics

Seven out of eight of the tested participant characteristics significantly predicted (main effect) total energy selected (Table 3, additional models). Parameter estimates (in models where non-significant interactions were removed) indicated that lower amounts of energy were selected by participants who were the most educated (−0.12, SE = 0.03, 99.5%IC [−0.21; −0.03]), with the highest income level (highest vs. lowest quintile: −0.20, SE = , 99.5%IC [−0.32; −0.08]), who were white (−0.18, SE = , 99.5%IC [−0.29; −0.07]), dieting (−0.30, SE = , 99.5%IC [−0.38; −0.22]), least hungry (0.006, SE = 0.001, 99.5%IC [0.004; 0.007]), most motivated by health (−0.08, SE = 0.01, 99.5%IC [−0.12; −0.05]) and motivated by controlling their weight (−0.05, SE = 0.01, 99.5%IC [−0.08; −0.02]). Participants of lower BMI tended to select lower amounts of energy (p = 0.009) but this did not reach the pre-registered threshold for statistical significance (p < 0.005). Across all of the models examining the moderating effects of participant characteristics (BMI, education level, household income, ethnicity/race, dieting status, baseline hunger, health and weight control motives) all interactions between labelling condition and participants characteristics were non-significant (Table 3, additional models). Therefore, although the presence of energy labelling and participant characteristics independently predicted energy selected, the effect of energy labelling on energy selected did not differ as a function of participant characteristics.

## 4. Discussion

Across twelve randomized control experiments we found that when participants made food and drink choices from menus that included energy labelling, the total energy content of selections was lower compared to the same menus without energy labelling. However, there was no evidence that this effect of energy labelling on food/drink choices differed across a range of participant demographics or characteristics.

The results of the present study are consistent with some systematic reviews and meta-analyses which suggest that the effect of energy labelling on energy content of food or drink selections is likely to be small in size (Robinson et al., 2021a). In the experiments included in the present synthesis, food and drink selections were mostly hypothetical. Because there is some indication that the effect of energy labelling on energy selected may be smaller when actual food choices are made in OOHF outlets (Cantu-Jungles et al., 2017), the present results may overestimate the effect that energy labelling has in real world settings. The associations we observed between participant demographics or characteristics and energy selected (independent of energy labelling) are consistent with some previous research which has examined energy content of food choices/eating occasions. For example, lower hunger and being female (as opposed to male) have been shown to be independently associated with consuming less energy during eating occasions (Robinson et al., 2017; Sadoul et al., 2014). Likewise, there is evidence that being more motivated by health or weight control when making food choices and being of higher socioeconomic position (SEP) are associated with lower energy intake and improved diet quality (Darmon & Drewnowski, 2008; Marty et al., 2021b). Yet, irrespective of there being patterning of energy associated with participant demographics or characteristics, none of these demographics/characteristics predicted the influence that energy labelling had on energy selected.

Understanding whether energy labelling has an equitable effect on consumer behaviour is of importance as it is key that interventions to improve diet and reduce obesity do not contribute to inequalities in health and benefit those most in need. Although some observational studies have found that self-reported use of energy labelling in the OOHF sector differs by demographic profile (e.g. females being more likely to report noticing and using labelling) (Chen et al., 2015), there is a lack of supporting evidence from randomized control trials. In relation to SEP, a 2015 review concluded that there was not convincing evidence that the impact of energy labelling impacts on food choice differed in lower vs. higher SEP groups, but highlighted the need for further study (Sarink et al., 2016). The present study does not support the proposition that there are participant demographics or characteristics that moderate the effect of energy labelling on the energy content of food and drink selections. Instead, our findings suggest that irrespective of participant demographics (including age, sex, ethnicity, education level, income) or characteristics (BMI, food choice motives, current hunger), energy labelling exerts a small influence on consumer behaviour.

The studies included in the present research predominantly involved participants making hypothetical food/drink choices (with the exception of a single study (Robertson & Lunn, 2020)) and this is an important limitation. In real-world settings in which selections are required to be paid for and then consumed findings may differ. For example, in a study of fast food chains in the US (Petimar et al., 2019) there was no evidence that a decrease in average energy purchased differed by participant income. However, after this initial decrease, there was an upward trend in average energy purchased over the next year and this trend was slightly more pronounced among lower as opposed to higher income participants. Thus, it may be the case that when food selections occur in the real-world, other factors such as concerns about price or value for money reduce the impact of energy labelling on food selections among lower income groups. Yet, in a different US study there was no evidence of effects of energy labelling on energy purchased differing based on customer income levels (Dumanovsky et al., 2011). It will be important for future research to examine whether the results of the present analyses generalise to contexts in which food selections are made under real-world conditions. It is also important to note that in real-world settings the implementation of energy labelling policies may result in businesses reformulating and reducing food product energy content (Zlatevska et al., 2018). Dependent on existing demographic patterning of purchasing behaviour, this may result in differing effects on energy consumed in OOHF settings. The present research does not account for this and future research will benefit from considering both consumer behaviour and business responses to energy labelling in conjunction.

Strengths of the present research include a large sample size, use of data from randomized control trials and pre-registration of analyses. A limitation of the present research is that effects of energy labelling were examined at a single time point. It will be valuable for future research to examine if results persist over time, as there is evidence that consumers partially compensate for reductions in energy intake by eating more later in the day (Robinson et al., 2022a, 2022b) It is also important to note that although we were able to sample a large and diverse group of participants, the majority were of higher education status. It may be the case that had we sampled a greater number of participants with more extreme levels of SES (e.g. no formal education or schooling, very low household income, experiencing food insecurity) results would differ. We were also limited by available data. Because of this we were unable to examine how energy labelling affects food choices among groups other than male vs. female and among specific ethnic minority groups. Future research should aim to address this. Finally, although a strength of the work is the large sample size of pooled analysis, we identified eligible studies by contacting a small number of research groups conducting research in the field and working collaboratively, as opposed to adopting a systematic review approach. Therefore, replication of the present findings using data from other studies and cultural contexts would be informative.

## 5. Conclusions

Energy labelling was associated with a small reduction in energy content of food selections and this effect was similar across a range of participant demographics and characteristics. These preliminary findings suggest that energy labelling policies may not produce or widen or narrow existing inequalities in diet.

## Supplementary Material

Online supplement

## Figures and Tables

**Table 1:** Included studies.

	Country	Setting & Sample	Outcome measure	Sex	Age	BMI	Education level	Income	Ethnicity/race	Dieting status	Food choices motivated by health	Food choices motivated by weight	Hunger	Sample size for present analyses

1)Marty 2020 Study 1 (Marty et al., 2020)	UK	Online, general population	Total kcals selected [hypothetical]	M vs F	Contin (>18yrs)	SR Contin	Categorical	Household	White vs not	Y vs N	Single item (1–7)	Single item (1–7)	−	868
2)Marty 2020 Study 2 (Marty et al., 2020)	UK	Online, general population	Total kcals selected [hypothetical]	M vs F	Contin (>18yrs)	SR Contin	Categorical	Household	White vs not	Y vs N	Single item (1–7)	Single item (1–7)	−	875
3)Marty 2021 Study 1 (Marty et al., 2021a)	US	Online, general population	Total kcals selected [hypothetical]	M vs F	Contin (>18yrs)	SR Contin	Categorical	Household	White vs not	Y vs N	Single item (1–7)	Single item (1–7)	0–100	1001
4)Marty 2021 Study 2 (Marty et al., 2021a)	US	Online, general population	Total kcals selected [hypothetical]	M vs F	Contin (>18yrs)	SR Contin	Categorical	Household	White vs not	Y vs N	6 items (1–4)	3 items (1–4)	0–100	1090
5)Tapper 2022 Study 1 (Tapper et al., 2022)	UK	In-person, campus sample	Total kcals selected [hypothetical]	M vs F	Contin (>18yrs)	M Contin	−	−	−	Y vs N	Single item (1–7)	Single item (1–7)	−	70
6)Tapper 2022 Study 2 (Tapper et al., 2022)	UK	In-person, campus sample	Total kcals selected [hypothetical]	M vs F	Contin (>18yrs)	M Contin	−	−	−	Y vs N	Single item (1–7)	Single item (1–7)	0–100	280
7)Masic 2017 (Masic et al., 2017)	UK	Online, general population	Total kcals selected [hypothetical]	M vs F	Contin (>18yrs)	SR Contin	Categorical	Household	White vs not	Y vs N	Single item (1–7)	Single item (1–7)	0–100	236
8)Robertson 2020 (Robertson & Lunn, 2020)	Ireland	In-person, general population	Total kcals selected [actual]	M vs F	Contin (>18yrs)	−	Categorical	−	−	−	−	−	−	71
9)Van Epps 2021 Study 3 (VanEpps et al., 2021)	US	Online, general population	Total kcals selected [hypothetical]	M vs F	Contin (>18yrs)	−	−	−	−	−	−	−	−	1219
10 Van Epps 2021 Online Study 2 (VanEpps et al., 2021)	US	Online, general population	Total kcals selected [hypothetical]	M vs F	Contin (>18yrs)	SR Contin	−	Household	−	Y vs N	−	−	−	1410
11)Van Epps 2021 Online Study 3 (VanEpps et al., 2021)	US	Online, university alumni sample	Total kcals selected [hypothetical]	M vs F	Contin (>18yrs)	SR Contin	All university educated	Household	−	Y vs N	−	−	−	672
12)Haynos 2017 (Haynos & Roberto, 2017)	US	Online, university sample	Total kcals selected [hypothetical]	F	Contin (>18yrs)	SR Contin	−	−	Multi-categories	Y vs N	−^a^	−^a^	0–100	716

Contin = continuous, SR = self-reported, M = measured.

a Included a measure of whether the specific food choice made during the study was motivated by weight control/health (state measurement of motivation), but as this was measure was not consistent with the other studies (e.g. extent to which every day food choices are motivated by weight control/health - trait measure of motivation), it was not included.

**Table 2:** Sample characteristics.

	N	Values

Age, *years*	8486 (12/12 studies)	
Mean (SD)		36.0 (14.8)
Categories, n (%)		
18–25		2433 (28.7)
26–35		2365 (27.7)
35–50		2175 (25.6)
>50		1524 (18.0)
Sex, *female*, n (%)	8464 (12/12 studies)	4863 (57.5)
BMI, *kg/m^2^*	6923 (10/12 studies)	
Mean (SD)		26.6 (6.51)
Categories, n (%)		
≤18.5		253 (3.7)
18.6–24.9		3081 (44.5)
25.0–29.9		2010 (29.0)
≥30		1579 (22.8)
Education level, n (%)	4575 (6/12 studies)	
Lower (no university degree)		1725 (37.7)
Higher (university degree)		2850 (62.3)
Ethnicity/race, n (%)	4620 (6/12 studies)	
White		3742 (81.0)
Non-white ethnic minorities		878 (19.0)
Dieting status, n (%)	7186 (10/12 studies)	
Dieting		1822 (25.3)
Not dieting		5364 (74.7)

**Table 3:** Mixed models testing the effect of energy labelling and participant characteristics on total kcal selected across the 12 studies (including random effect of study).

	N	Type III Tests	
		F value	p value

Primary model – step 1	8460 (12/12 studies)		
Labelling		34.36	<0.001
sex		122.59	<0.001
age Primary model – step 2	8460 (12/12 studies)	62.49	<0.001
labelling*sex		0.14	0.709
labelling*age			
Primary model (raw) –	8460 (12/12 studies)	0.94	0.419
step 1			
labelling		19.43	<0.001
sex		33.33	<0.001
age Primary model (raw) –	8460 (12/12 studies)	27.32	<0.001
step 2			
labelling*sex		0.01	0.976
labelling*age Additional model 1^a^	6901 (10/12 studies)	0.17	0.916
BMI		3.90	0.009
labelling*BMI Additional model 2	4544 (6/12 studies)	1.21	0.306
education		14.47	<0.001
labelling*education Additional model 3	5857 (6/12 studies)	0.42	0.516
Income		8.84	<0.001
labelling*income Additional model 4	4603 (6/12 studies)	1.15	0.329
Ethnicity/race		22.53	<0.001
labelling*ethnicity/race Additional model 5	7163 (10/12 studies)	0.06	0.811
Dieting		116.17	<0.001
labelling*dieting Additional model 6	3305 (5/12 studies)	0.01	0.914
Hunger		73.83	<0.001
labelling*hunger Additional model 7	4168 (6/12 studies)	0.37	0.542
health motives		47.78	<0.001
weight motives		21.14	<0.001
labelling*health motives		0.33	0.565
labelling*weight		0.64	0.424
motives			
Sensitivity model 1 –	8460 (12/12 studies)		
step 1			
labelling		34.91	<0.001
sex		126.74	<0.001
age (continuous)			
Sensitivity model 1 –	8460 (12/12 studies)	208.87	<0.001
step 2			
labelling*sex		0.20	0.657
labelling*age		2.47	0.116
(continuous)			
Sensitivity model 2 –	8225 (11/12 studies – excluding Masic et al.)		
step 1			
labelling		29.80	<0.001
sex		116.83	<0.001
age Sensitivity model 2 –	8225 (11/12 studies – excluding Masic et al.)	62.40	<0.001
step 2			
labelling*sex		0.44	0.505
labelling*age			
Sensitivity model 3 –	7746 (11/12 studies – excluding female only study)	0.76	0.518
step 1			
labelling		34.81	<0.001
sex		118.94	<0.001
age Sensitivity model 3 –	7746 (11/12 studies – excluding female only study)	61.60	<0.001
step 2			
labelling*sex		0.08	0.778
labelling*age			
Sensitivity model 4 –	7806 (11/12 studies – excluding university degree only study)	0.78	0.502
step 1			
labelling		30.89	<0.001
sex		103.69	<0.001
age Sensitivity model 4 –	7806 (11/12 studies – excluding university degree only study)	52.99	<0.001
step 2			
labelling*sex		0.30	0.582
labelling*age			
Sensitivity model 5^a^	6901 (10/12 studies)	1.79	0.147
BMI (continuous)		20.98	<0.001
labelling*BMI		1.39	0.238
(continuous)			

a All additional models and sensitivity model 5 include the mentioned additional variables in the primary model – step 1, i.e. labelling, sex, age and additional variables.

## Data Availability

Data is deposited online

## Footnotes

1 We compared AIC and BIC of the primary model before and after the inclusion of a random effect of study and found smaller AIC and BIC in the model including the random effect (AIC = 23688, BIC = 23689) which indicated an improved model fit compared to the model without the random effect (AIC = 23690, BIC = 23697).
