# Supplementary material for: Is the effect of menu energy labelling on consumer behaviour equitable? A pooled analysis of twelve randomized control experiments
Source: Appetite. Author manuscript; Available in PMC 2024 Mar 1. (PMC10082393; doi:10.1016/j.appet.2023.106451)
Supplement: Online supplement [file NIHMS1884265-supplement-Online_supplement.docx]

Online Supplementary Materials

*Standardising of measures*

Health-based food choice motivation was available as a 1-7 scale in 5/6 of the studies and a 1-24 scale in the remaining study. We multiplied the total score on the 1-24 scale by 0.2916 and rounded to the nearest whole number in order to produce a comparable 1-7 scale. Weight control-based food choice motivation was available as a 1-7 scale in 5/6 of the studies and a 1-12 scale in the remaining study. We multiplied the 1-12 scale score by 0.5833 and rounded to the nearest whole number in order to produce a comparable 1-7 scale.

*Statistical significance and power*

Due to the large number of comparisons and models being conducted (interactions between 10 predictor variables and labelling condition, 0.05/10), alpha will be set at p < .005 across all analyses. Our main analysis approach involves examining whether the inclusion of a participant individual difference variable and its interaction term with labelling condition into the basic model results in a significant increase in variance explained. To detect a statistically small increase in variance explained in linear regression (GPower 3.1.9.7 [linear multiple regression, R^2^ increase], f^2^ = 0.02, 80% power, p < .005, largest plausible tested model = 15 predictor variables, 4 variables tested in second step of the model), a minimum sample size of n=944 would be required. As all our models from the main analyses are expected to comfortably exceed this sample size (see above), we are well powered to detect statistically small interactions between the effect of kcal labelling and participant characteristics. It is difficult to estimate the power ramifications for the inclusion of the random effect of study (if model fit is improved). However, the number of levels of the random effect of study (i.e. 12 studies) exceeds the commonly used cut-off for a minimum of 5 levels the inclusion of the random effect would be unlikely to greatly bias or change parameter estimates for the variables of interest in our analyses (Gomes, 2021). In the present analyses the inclusion of the random effect is to account for variability in the outcome variable that is unattributed to kcal labelling and participant characteristics, as opposed to any hypotheses or inferences being made about the random effects term. Therefore, we presume that if the inclusion of a random effect of study does improve model fit, then statistical power to detect fixed effects of kcal labelling (and participant characteristic moderation) will be similar to statistical power for the default model (as described above) in which there is no statistical justification (i.e. improved model fit) to include a random effect of study.

*References*

Gomes DGE. Including random effects in statistical models in ecology: fewer than five levels? bioRxiv. 2021:2021.04.11.439357.

Raw data from studies for primary model (raw) analysis

|  | **Control** | | | **Labelling** | | | **% reduction** |
| --- | --- | --- | --- | --- | --- | --- | --- |
|  | n | mean kcal | sd | n | mean kcal | sd |  |
| Study 1 | 421 | 888.4 | 211.9 | 447 | 874.3 | 222.3 | -1.58 |
| Study 2 | 428 | 920.3 | 238.3 | 447 | 898.7 | 233.0 | -2.35 |
| Study 3 | 497 | 1718.5 | 437.8 | 504 | 1618.9 | 442.3 | -5.80 |
| Study 4 | 546 | 1606.2 | 408.1 | 544 | 1538.2 | 425.8 | -4.23 |
| Study 5 | 34 | 535.3 | 277.5 | 35 | 622.7 | 323.2 | 16.33 |
| Study 6 | 139 | 552.9 | 360.4 | 141 | 552.6 | 358.1 | -0.05 |
| Study 7 | 121 | 1998.0 | 236.4 | 115 | 1911.4 | 250.4 | -4.34 |
| Study 8 | 48 | 866.1 | 444.8 | 23 | 702.7 | 391.2 | -18.86 |
| Study 9 | 611 | 986.5 | 395.7 | 608 | 900.8 | 352.8 | -8.69 |
| Study 10 | 938 | 853.6 | 336.0 | 472 | 841.7 | 326.6 | -1.39 |
| Study 11 | 449 | 677.7 | 230.9 | 223 | 660.1 | 230.5 | -2.61 |
| Study 12 | 360 | 1462.5 | 1210.6 | 356 | 1380.7 | 1564.6 | -5.59 |
|  |  |  |  |  |  | **Weighed mean% reduction** | **-3.93** |
